# 10-hydroxy-2-decenoic acid of royal jelly exhibits bactericide and anti-inflammatory activity in human colon cancer cells

**DOI:** 10.1186/s12906-018-2267-9

**Published:** 2018-07-03

**Authors:** Yuan-Chang Yang, Wing-Ming Chou, Debora Arny Widowati, I-Ping Lin, Chi-Chung Peng

**Affiliations:** 1 Department of Biotechnology, National Formosa University, Huwei District, Yunlin Taiwan; 2 Department of Research and Development, Challenge Bioproducts Co., Ltd., Yunlin, Taiwan

**Keywords:** 10-hydroxy-2-decenoic acid, Anti-inflammation, Antimicrobial, Colon cancer cell, Cytokines

## Abstract

**Background:**

Royal jelly (RJ), the exclusive food for the larva of queen honeybee, is regarded as the novel supplement to promote human health. The function of RJ may be attributed to its major and unique fatty acid, 10-hydroxy-2-decenoic acid (10-HDA). The current study investigated the anti-inflammory function of 10-HDA on human colon cancer cells, WiDr, as well as its effect on the growth of pathogenic bacterium.

**Methods:**

The pro-inflammatory cytokines, receptor antagonist cytokine (IL-1ra) and nuclear factor-kappa B (NF-κB) in WiDr cells was analyzed by Enzyme-linked immunosorbent assay (ELISA) or western blot. The growth inhibition of 10-HDA on bacterium was evaluated by determination of minimal inhibitory concentrations (MIC) and minimal bactericide concentrations (MBC).

**Results:**

The production of pro-inflammatory cytokines, Interleukin (IL)-8, IL-1β and tumor necrosis factor-alpha (TNF-α) in WiDr cells was modulated by 10-HDA. IL-8 were dramatically declined by 10-HDA at 3 mM, while IL-1β and TNF-α were significantly decreased. 10-HDA increased IL-1ra in a dose manner. NF-κB pathway is primarily in response to prototypical pro-inflammatory cytokines, and NF-κB was reduced after 10-HDA treatment. 10-HDA acted as potent bactericide against animal- or human-specific pathogens, including *Staphylococcus aureus, Streptococcus alactolyticus, Staphylococcus intermedius B, Staphylococcus xylosus, Salmonella cholearasuis, Vibro parahaemolyticus* and *Escherichia coli (hemolytic*).

**Conclusions:**

The current study showed that in vitro 10-HDA from RJ exhibited anti-inflammatory activity in WiDr cells, as well as anti-bacterial activity against animal pathogens. 10-HDA showed its potential as anti-imflammtory agent and bactericide to benefit human gastrointestinal tract.

## Background

In response to pathogens, the pro-inflammatory cytokines, TNF-α is induced by activated macrophages; subsequently, the spread of pathogens into the circulation is limited [1, 2]. TNF-α initiates a cascade of pro-inflammatory cytokines [3]. Among them, IL-1 a primary pro-inflammatory cytokine stimulates the expression of genes that are associated with inflammation [4]. IL-8 is rapidly induced by IL-1 or TNF-α, and used as a marker of activated inflammatory/immuno response [5]. However, the production of IL-1 is inhibited by an excess of IL-1ra [6]. The canonical NF-κB pathway has been defined primarily in response to TNF-α and IL-1 signaling, prototypical proinflammatory cytokines. The persistent, long-lasting inflammation may lead to inflammatory disease and cancer etiology [7]. The over-production of pro-inflammatory cytokines and NF-κB take important roles in the chronical inflammatory diseases such as rheumatoid arthrtitis (RA) and inflammatory bowel disease (IBD) [8]. Inflammation is generally treated with immune-suppressing drugs that reduce inflammation and painful symptoms. It was reported that non-steroidal anti-inflammatory drugs might cause the gastrointestinal complications [9]. It is thus needed to explore the natural substances that inhibit inflammation and prevent chronic inflammatory diseases with minimal toxicity [10].

Royal jelly (RJ), secreted by the hypopharyngeal and mandibular glands of worker honeybees (*Apis mellifera*), is supplied as the exclusive food for the larva and adult of queen honeybee. RJ well known for its novel functions to promote human health mainly comprises water, sugar, proteins, and lipids. The lipid is present in around 3 to 19% of dry RJ [11]. And approximately 90% of RJ lipids are free fatty acids, containing 8–12 carbons that are usually either hydroxyl or dicarboxylic forms. The major and unique fatty acid in RJ lipid is 10-hydroxy-2-decenoic acid (10-HDA), which was not found in other bee products [12].

The pharmacological activities of RJ were reported, including growth rate increasing [13], antitumor [14, 15], anti-inflammatory and antimicrobial activity [16–19], as well as antioxidant activity [20]. RJ has inhibitory effects toward approximately 30 bacterial species, including aerobes, anaerobes, Gram-positive and Gram-negative bacteria [21]. 10-HDA is one of the compositions responsible for the pharmacological activities of RJ. It was reported to have anti-tumor [22], collagen promoting [23], immunomodulatory [24], antimicrobial activity [16–18] and antimelanogensis [25].

In this study, we investigated the effect of 10-HDA purified from RJ on human colon adenocarcinoma cell (WiDr cells), as well as antimicrobial activity against serveral animal pathogenic bacteria. The supression of TNF-α, IL-1β, IL-8 and NF-κB pathway by 10-HDA was evaluated, as well as the induction of IL-1ra.

## Methods

### Preparation of 10-HDA from royal jelly

Royal jelly was provided by the Fu-Chang Beekeeping in Hualien, Taiwan. The 10-HDA was purified and quantitative analysis using the method from our previous work [25]. The highest purity of the 10-HDA sample was obtained about 92%. This sample was used on WiDr cells for the next assays.

### Cell viabilities assay

The WiDR human adenocarcinoma cell (BCRC 60157) was cultured in minimum essential medium (Eagle) with 2 mM L-glutamine and Earle’s BSS adjusted to contain 1.5 g/L sodium bicarbonate, 1.0 mM sodium pyruvate, 90%; fetal bovine serum, 10%, at 37 °C in a humidified atmosphere containing 5% (*v*/v) CO_2._ To study the effect of 10-HDA on WiDr cell proliferation, the cells were seeded in 96-well plates at a density of 1 × 10^4^ cells/well. After one night, the cells were then treated with different doses of 10-HDA, ranging from0.1 to 5 mM, for 24 h. After the 24-h treatment, the supernatant were collected. Cell viability was evaluated by the MTT (methyl thiazol tetrazolium bromide) assay as described previously [25]. The optical density was determined at 570 nm (OD_570_).

### Cytokines analysis

The cytokines production of TNF-α, IL-1β, IL-8 and IL-1ra in the cell culture supernatants collected after 24 h of cultivation were determined by ELISA kit (DUO Set, R&D systems, Abingdon, UK) according to the manufacturer’s instructions. Capture antibody (1:180 dilution) was added into each well of a 96-well microplate, incubated overnight at room temperature. In the following day, after washing, block buffer was added and incubated for 1 h. The wells were washed again, then the standards and the culture supernatant of the 10-HDA-treated cells was added and incubated at room temperature for 2 h. After washing, the detection antibody (1:180 dilution) was added to each well. After incubated for 2 h, Streptavidin horseradish-peroxidase (HRP: reagent diluent = 1: 200) was added, then kept away from light for 20 min. Finally, substrate solution was added to each well, kept for 20 min before adding 50 μL stop solution to each well. The optical density was determined at 450 nm (OD_450_).

### NF-κB western assays

The NF-κB production in the cell were determined by western blot as described previously [8]. The cells were lysed in RIPA buffer (pH 7.4, 50 mM tris, 0.1% SDS, 50 mM NaCl, 1% NP-40, 1 mM PMSF, 10 μg/mL aprotinin and 10 μg/mL leupeptin) and collected its nuclear fraction. Protein quantified by Bradford assay using BSA as the standard and the proteins were separated by 10% SDS-PAGE, transferred onto hybond-C Extra nitrocellulose membrane (Amersham Biotscience, U.K.). The membranes were blocked overnight in block solution (TBS-T buffer containing 5% non-fat skim milk). The membrane was then incubated with rabbit polyclonal anti-NF-κB and antiβ-actin antibodies as an internal control. The membranes were further incubated with HRP conjugated anti-rabbit goat polyclonal secondary antibody. Proteins were detected by chemilluminescent with Super Signal® West Pico Chemiluminescent Substrate (ECL) (Thermo Scientific). The signal density of each band was measure and analyzed using a densitometer system Gel Doc TM / Chemi Doc TM Universal hood II (Bio-Rad).

### Antimicrobial activity assay

Antimicrobial activity were evaluated by against four Gram-positive bacteria, *Staphylococcus aureus, S. intermedius B, S. xylosus,* and *Streptococcus alactolyticus*, and four Gram-negative bacteria, *hemolytic Escherichia coli, Pseudomonas aeruginosa, Salmonella cholearasuis,* and *Vibrio parahaemolyticus* [19]. The MIC and MBC of 10-HDA was measured using the method according our previous work [26]. 10-HDA was dissolved in dimethyl sulphoxide (DMSO) at a final concentration of 100 μM. The assay solution contain 100 μl of 10-HDA dilution, 100 μl of culture medium (Tryptone Soya Broth (TSB)) and 100μl of a 10× suspension of each microorganism in 96-well microtiter plate (Iwaki Inc., Japan). Each assay and growth control well was inoculate with final concentration of a bacterial is 1–5 × 10^5^ CFU/well. Bacterial growth was detected by optical density (OD) (ELISA reader, μQuant microplate spectroplate, Bio-TEK, VT, USA).

### Statistical analysis

Data are presented as mean ± SD. Statistical analysis was performed using SigmaPlot software (Systat Software Inc., San Jose, CA, USA). Significant differences were evaluated using one way ANOVA followed by the Dunnett’s multiple comparison test. Values of *p* ≤ 0.05 or less were considered as statistically significance.

## Results

### 10-HDA inhibited growth of WiDr cells

**Fig. 1:**
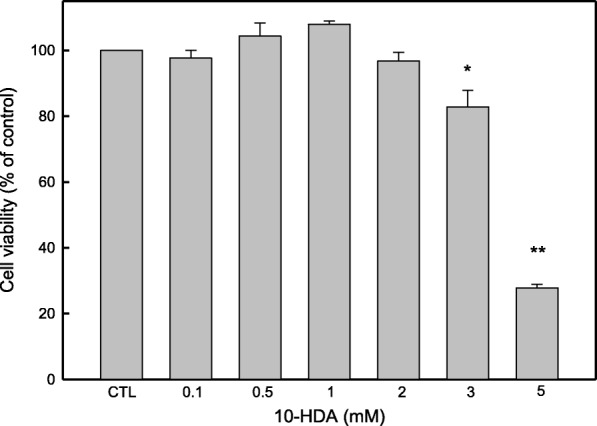
Percent cell viability of WiDr colon cancer cells after 10-HDA treatment (0.1, 0.5, 1, 2, 3, and 5 mM) for 24 h. CTL represents the control group (without 10-HDA). Each bar is a mean ± SD of the triplicates from one representative experiment. * = *p* ≤ 0.05; ** = *p* ≤ 0.01 compared to control

The MTT assay was performed to assess the rate of proliferation of WiDr human colon cancer cells after treatment with various concentrations of 10-HDA (0.1–5 mM). Fig. 1 shows that the inhibitory effect of 10-HDA on WiDr cell proliferation is dose-dependent. The inhibitory effects of 10-HDA were more pronounced at the higher dose (5 mM), which has cytotoxic effect and could kill the WiDr cells directly. 10-HDA at a concentration of 3 mM inhibited significantly about 82.82% of the cell proliferation compared to the control group as the 100%, but it was not cytotoxic to the WiDr cells. Lower doses of 10-HDA did not inhibited the cells significantly. The maximum growth inhibitory effect of 10-HDA was observed at the 5 mM concentration after 24 h of treatment.

### 10-HDA inhibited TNF-α, IL-1β and IL-8 production in WiDr cells

**Fig. 2:**
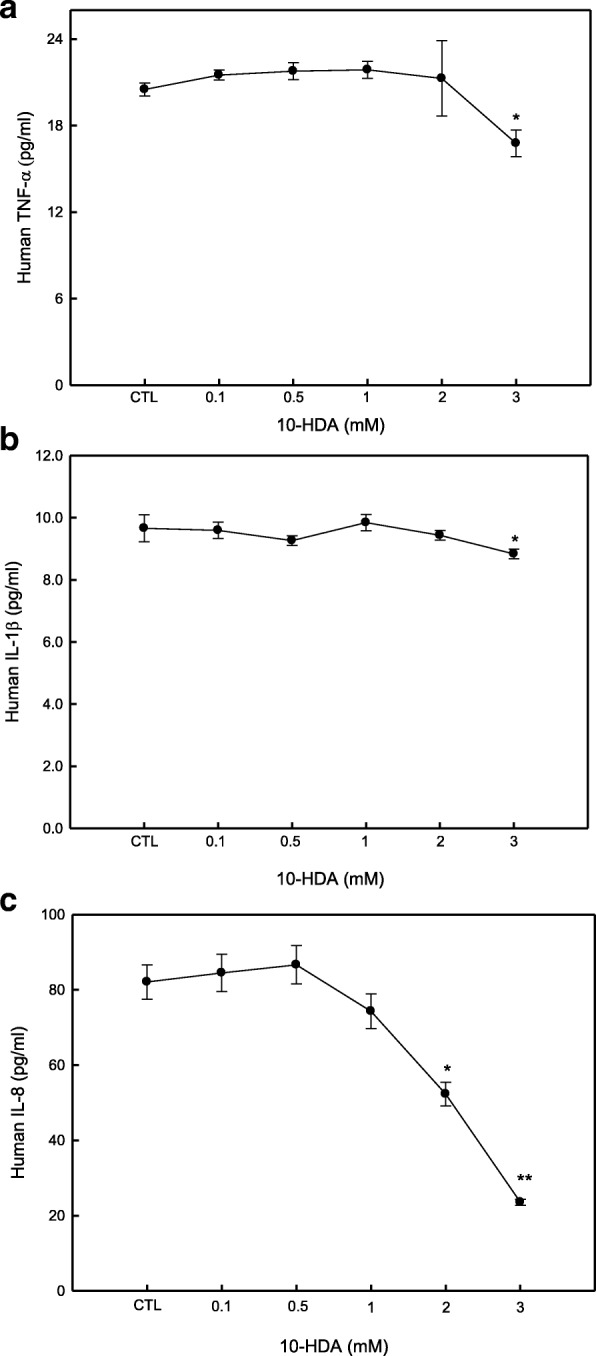
Effect of 10-HDA on human TNF-α, IL-1β and IL-8 production in WiDr cells. Supernatants of WiDr cultures, treated with or without 10-HDA (0.1, 0.5, 1, 2 and 3 mM), were collected after 24 h and tested by ELISA for detecting TNF-α (**a**), IL-1β (**b**) and IL-8 (**c**). CTL represents the control group (without 10-HDA). Each point is a mean ± SD of the triplicates from one representative experiment. * = *p* ≤ 0.05; ** = *p* ≤ 0.01 compared to control

We investigated the effects of 10-HDA on TNF-α, IL-1β, IL-8 and IL-1ra production in WiDr cells by ELISA. After examining the effec of 10-HDA on human TNF-α production, it was observed that 10-HDA inhibited TNF-α at a concentration of 3 mM 10-HDA, about 81.79% compared to the control. The level of TNF-α secreted by the control group was about 20.50 pg/mL, whereas in the group treated with a concentraion of 10-HDA at 3 mM, the TNF-α secretion level was reduced to 16.76 pg/mL (Fig. 2a). Further results indicated that 10-HDA inhibited human IL-1β secretion at 24 h in WiDr cells. The WiDr cells in the control group produced 9.66 pg/mL IL-1β, but the IL-1β secretion decreased to 8.83 pg/mL when the cells were treated with a concentration of 10-HDA at 3 mM (Fig. 2b). Fig. 2c shows that 10-HDA markedly inhibited IL-8 secretion at non-cytotoxic concentrations (2 and 3 mM) at 24 h treatment. In the control group, the WiDr cells produced 82.08 pg/mL IL-8, and the treatement in 2 mM and 3 mM 10-HDA reduced the human IL-8 production by 72.31 and 43.57%, respectively.

### 10-HDA inhibited NF-κB expression in WiDr cells

**Fig. 3:**
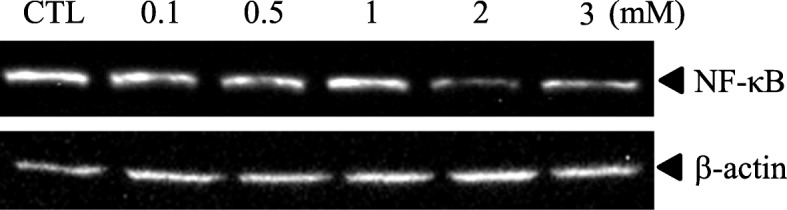
Effect of 10-HDA on human NF-κB production in WiDr cells. Supernatants of WiDr cultures, treated with or without 10-HDA (0.1, 0.5, 1, 2 and 3 mM), were collected after 24 h and tested by Western Blotting to detect the presence of NF-κB. CTL represents the control group (without 10-HDA). Results are expressed as relative percentages of NF-κB activation compared to the control group (100%)

To investigate the effect of 10-HDA on NF-κB expression, we examined this effect using WiDr cells. The result (Fig. 3) was observed that 10-HDA inhibited the NF-κB expression, the inhibition rate were approximately ranging from 6.56 to 68.9% compared to the control. This result suggested that 10-HDA would modulate the inflammatory response in a dose-dependent manner.

### 10-HDA induced IL-1ra production in WiDr cells

**Fig. 4:**
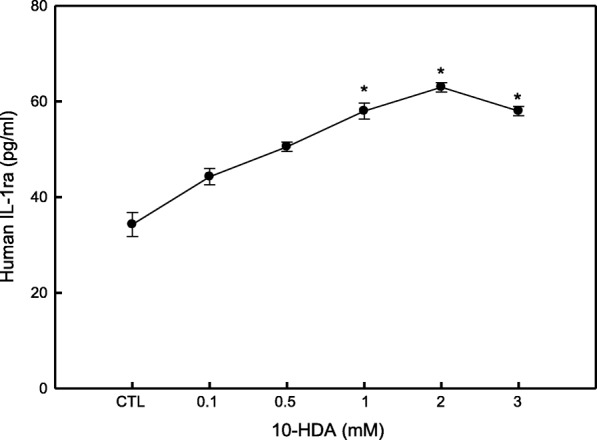
Effect of 10-HDA on human IL-1ra production in WiDr cells. Supernatants of WiDr cultures, treated with or without 10-HDA (0.1, 0.5, 1, 2, 3, and 5 mM), were collected after 24 h and tested by ELISA for detecting IL-1ra. CTL represents the control group (without 10-HDA). Each point is a mean ± SD of the triplicates from one representative experiment. * = *p* ≤ 0.05 compared to control

IL-1ra is one of the major anti-inflammatory cytokine [27]. The results on Fig. 4 indicated that 10-HDA stimulated the human IL-1ra production in WiDr cells. 10-HDA concentration at 1, 2 and 3 mM showed the significant stimulation activity on the IL-1ra secretion (57.97, 62.95, and 57.97 pg/mL, respectively) compared to the control, in which the WiDr cells only produced 34.26 pg/mL of IL-1ra.

### Antibacterial activity of 10-HDA

10-HDA was assayed for bactericidal and bacteriostatic activities against eight strains of Gram-positive and Gram-negative bacteria (Table 1). Result showed that the MIC of 10-HDA against Gram-positive bcateria was 23–44 μM, and MBC was 33–66 μM. The MIC and MBC of 10-HDA against Gram-negative bcateria were in the ranges of 40–43 μM and 74–78 μM, respectively. However, the growth of the Gram-negative bcateria, *Pseudomonas aeruginosa*, was not affected by 10-HDA at all*.* 10-HDA has higher antibacterial activity against most of the Gram-positive bacteria as well as several Gram-negative bacteria.

**Table 1:** Bacteriostatic and Bactericidal Activities of 10-HDA against Eight Different Animal- and Human-Specific Pathogens

Bacteria	MIC (μM)	MBC (μM)
*Staphylococcus aureus (+)*	23	53
*Streptococcus alactolyticus(+)*	44	66
*Staphylococcus intermediusB(+)*	23	33
*Staphylococcus xylosus(+)*	24	36
*Pseudomonas aeruginosa(−)*	NI	ND
*Salmonella cholearasuis(−)*	42	74
*Vibro parahaemolyticus(−)*	40	76
*Escherichia coli (hemolytic)*	43	78

*NI: No inhibition activity

*ND: Not Detected

## Discussion

In this study, 10-HDA was showed to inhibit the production of pro-inflammatory cytokines, TNF-α, IL-1β and IL-8 in WiDr cells. In contrast, 10-HDA effectively induced the production of IL-1ra at a dose, from 0.1 to 3 mM. Consequently, abundant of IL-1ra restrained the production of IL-1β. The production of IL-8 was siginficantly reduced in a dose-dependent manner at a dose, from 0.5 to 3.0 mM of 10-HDA. 10-HDA inhibited the production of NF-κB in WiDr cells as well. In summany, 10-HDA exhibited anti-inflammation function in WiDr cells. To our knowledge, it is the first time to show that 10-HDA also has in vitro anti-inflammory activity in the human colon cancer cells.

10-HDA was reported to have pharmacological functions potentially due to its anti-tumor [28–30], angiogenesis–inhibition [22], and immunomodulatory activities [31]. The pro-inflammatory cytokines such as TNF-a, IL-1β, IL-8 and TGF-β may trigger inflammatory diseases.10-HDA could act as Histone deacetylase inhibitor (HDACI) to inhibit the proliferation of FLS cells from RA, a systemic chronic inflammatory disease [31]. HDACIs have emerged as potent anti-inflammatory drugs to treat inflammatory diseases [32].

The chronic inflammation is considered as a major cause in cancer etiology [7]. Either IL-8 or IL-1 could stimulate the proliferation of melanoma, pancreatic carcinoma and colon carcinoma cell lines [33]; while IL-1ra could inhibit tumor growth [20]. It was reported that serum IL-1ra were reduced in colorectal cancer patients [34]. Inhibition of NF-κB expression is one of strategies to prevent carcinogenesis. 10-HDA inhibited the production of TNF-α, IL-1β, IL-8, and NF-κB in WiDr cells; whereas it increased the amount of IL-1ra. Hence, 10-HDA perhaps can be supplied as a chemopreventive agent for chronic inflammation and carcinogenesis. Nevertheless, more study should be done to address whether 10-HDA has in vivo anti-inflammatory and anti-tumor activities in human gastrointestinal tract.

RJ possesses antimicrobial activity attributing to its antimicrobial peptide and fatty acids [35]. The potency of anti-bacterial properties of RJ could be from its unique fatty acid, 10-HDA [17, 35]. The results revealed that 10-HDA has high anti-bacterial activity toward animal- and human-specific pathogens, including *S. aureus, S. alactolyticus, S. intermedius B, S. xylosus, S.cholearasuis, V. parahaemolyticus* and *E. coli (hemolytic*). 10-HDA, one of the active compounds in RJ, is responsible for the novel functions of RJ. And more studies should be conducted to verify that 10-HDA may benefit our gastrointestinal tract from chronic inflammation and pathogen infection.

## Conclusion

The production of pro-inflammatory cytokines, TNF-α, IL-1β and IL-8 in WiDr cells was inhibited by 10-HDA. In contrast, 10-HDA effectively induced the production of IL-1ra at a dose, from 0.1 to 3 mM. The production of IL-8 was significantly reduced in a dose-dependent manner at a dose, from 0.5 to 3.0 mM of 10-HDA. Abundant of IL-1ra subsequently suppressed the production of IL-1β, which was reduced after 10-HDA treatment at 3 mM. 10-HDA inhibited NF-κB in WiDr cells as well. 10-HDA also possessed high anti-bacterial activity against animal pathogens. These results suggested that 10-HDA in RJ could benefit human gastrointestinal tract via its anti-inflammatory and anti-bacterial activities.

## Abbreviations

10-HDA: 10-hydroxy-2-decenoic acid
ELISA: Enzyme-linked immunosorbent assay
IBD: inflammatory bowel disease
IL-1ra: receptor antagonist cytokine
MBC: minimal bactericide concentrations
MIC: minimal inhibitory concentrations
MTT: methyl thiazol tetrazolium bromide
NF-κB: nuclear facctor-kappa B
RJ: royal jelly
TNF-α: tumor necrosis factor-alpha
TSB: Tryptone Soya Broth
